# Association of Interarm Systolic Blood Pressure Difference with Atherosclerosis and Left Ventricular Hypertrophy

**DOI:** 10.1371/journal.pone.0041173

**Published:** 2012-08-23

**Authors:** Ho-Ming Su, Tsung-Hsien Lin, Po-Chao Hsu, Chun-Yuan Chu, Wen-Hsien Lee, Szu-Chia Chen, Chee-Siong Lee, Wen-Chol Voon, Wen-Ter Lai, Sheng-Hsiung Sheu

**Affiliations:** 1 Division of Cardiology, Division of Nephrology, Department of Internal Medicine, Kaohsiung Medical University Hospital, Kaohsiung Medical University, Kaohsiung, Taiwan; 2 Division of Nephrology, Department of Internal Medicine, Kaohsiung Medical University Hospital, Kaohsiung Medical University, Kaohsiung, Taiwan; 3 Department of Internal Medicine, Kaohsiung Municipal Hsiao-Kang Hospital, Kaohsiung Medical University, Kaohsiung, Taiwan; 4 Faculty of Mecicine, College of Medicine, Kaohsiung Medical University, Kaohsiung, Taiwan; Ochsner Health System, United States of America

## Abstract

An interarm systolic blood pressure (SBP) difference of 10 mmHg or more have been associated with peripheral artery disease and adverse cardiovascular outcomes. We investigated whether an association exists between this difference and ankle-brachial index (ABI), brachial-ankle pulse wave velocity (baPWV), and echocardiographic parameters. A total of 1120 patients were included in the study. The bilateral arm blood pressures were measured simultaneously by an ABI-form device. The values of ABI and baPWV were also obtained from the same device. Clinical data, ABI<0.9, baPWV, echocariographic parameters, and an interarm SBP difference ≥10 mmHg were compared and analyzed. We performed two multivariate forward analyses for determining the factors associated with an interarm SBP difference ≥10 mmHg [model 1: significant variables in univariate analysis except left ventricular mass index (LVMI); model 2: significant variables in univariate analysis except ABI<0.9 and baPWV]. The ABI<0.9 and high baPWV in model 1 and high LVMI in model 2 were independently associated with an interarm SBP difference ≥10 mmHg. Female, hypertension, and high body mass index were also associated with an interarm SBP difference ≥10 mmHg. Our study demonstrated that ABI<0.9, high baPWV, and high LVMI were independently associated with an interarm SBP difference of 10 mmHg or more. Detection of an interarm SBP difference may provide a simple method of detecting patients at increased risk of atherosclerosis and left ventricular hypertrophy.

## Introduction

A blood pressure difference between arms is frequently encountered in various general populations [1]. This phenomenon, the “interarm difference” was first recognized more than 100 years ago [2]. “Blood pressure should be initially measured in both arms as patients may have large differences between arms. The arm with the higher values should be used for subsequent measurements” was suggested by current guidelines for the management of hypertension [3]. An appreciation of the presence of an interarm difference is vital for the accurate diagnosis and management of hypertension.

Recently, several studies have reported that a difference in systolic blood pressure (SBP) of 10 mmHg or more was strongly associated with subclavian stenosis (two cohorts; risk ratio [RR], 8.8; 95% confidence interval [CI], 3.6 to 21.2), peripheral vascular disease (five cohorts; RR, 2.4; 95% CI, 1.3 to 3.9), and pre-existing coronary artery disease (RR, 2.7; 95% CI, 1.8 to 3.9) [4], [5]. Furthermore, an interarm difference of 10 mmHg or more in SBP was strongly associated with increased cardiovascular mortality and all-cause mortality [6]–[8]. Compared with a difference in SBP of <10 mmHg, patients with a difference of ≥10 mmHg had increased cardiovascular (adjusted hazard ratio [HR], 4.2; 95% CI, 1.7 to 10.3) and overall mortality (adjusted HR, 3.6; 95% CI, 2.0 to 6.5) [8]. Data suggested that an interarm SBP difference, like peripheral artery disease indicated by a reduced ankle-brachial index (ABI), suggest poor prognosis [7], [9]. However, the exact mechanism between an interarm SBP difference and cardiovascular outcomes remains unclear.

The ABI is a simple, non-invasive, and reliable diagnostic tool for peripheral artery occlusive disease and an ABI<0.9 has been used to identify this condition in both clinical practice and epidemiologic studies [10]–[12]. The brachial-ankle pulse wave velocity (baPWV) has been reported as a good marker for arterial stiffness [13], [14]. A lower ABI and high baPWV show strong powers in predicting the mortality in various populations [15]–[18]. Moreover, baPWV is useful to screen a high-risk population in patients with ABI greater than 0.9 [16]. An interarm SBP difference is known to be associated with low ABI, but little is known about the relationship between an interarm SBP difference of 10 mmHg or more and baPWV and echocardiographic parameters. Accordingly, the aim of this study, using a technique of simultaneous blood pressure measurement, is to compare the ABI<0.9, baPWV, and echocardiographic parameters between patients with and without an interarm SBP difference of 10 mmHg or more and to identify the independent factors associated with an SBP interarm difference of 10 mmHg or more.

## Subjects and Methods

### Study Patients and Design

Study subjects were randomly included from a group of patients who arranged for echocardiographic examinations at Kaohsiung Municipal Hsiao-Kang Hospital. Patients with significant aortic or mitral valve disease, atrial fibrillation, or inadequate image visualization were excluded. We did not include all patients consecutively because baPWV, ABI, and blood pressures must be measured within 5 min after the completion of an echocardiographic examination. A total of 1120 patients (mean age 60.8±13.7 years, 636 males/484 females) were included.

### Ethics Statement

The study protocol was approved by the institutional review board of the Kaohsiung Medical University Hospital (KMUH-IRB-20120096). Informed consents have been obtained in written form from patients and all clinical investigation was conducted according to the principles expressed in the Declaration of Helsinki. The patients gave consent for the publication of the clinical details.

### Evaluation of cardiac structure and function

The echocardiographic examination was performed by one experienced cardiologist with a VIVID 7 (General Electric Medical Systems, Horten, Norway), with the participant respiring quietly in the left decubitus position. The cardiologist was blind to the other data. Two-dimensional and two-dimensionally guided M-mode images were recorded from the standardized views. The Doppler sample volume was placed at the tips of the mitral leaflets to obtain the left ventricular inflow waveforms from the apical 4-chamber view. All sample volumes were positioned with ultrasonic beam alignment to flow. Pulsed tissue Doppler imaging was obtained with the sample volume placed at the lateral corner of the mitral annulus from the apical 4-chamber view. The wall filter settings were adjusted to exclude high-frequency signals and the gain was minimized. The echocardiographic measurements included left ventricular internal diameter in diastole (LVIDd), left ventricular posterior wall thickness in diastole (LVPWTd), interventricular septal wall thickness in diastole (IVSTd), E-wave deceleration time, transmitral E wave velocity (E), transmitral A wave velocity, and early diastolic mitral velocity (Ea). Left ventricular systolic function was assessed by left ventricular ejection fraction (LVEF). Left ventricular mass was calculated using Devereux-modified method, i.e. left ventricular mass = 1.04×[(IVSTd+LVIDd+LVPWTd)^3^ – LVIDd^3^] – 13.6 g [19]. Left ventricular mass index (LVMI) was calculated by dividing left ventricular mass by body surface area. Left ventricular hypertrophy (LVH) was defined as suggested by the 2007 European Society of Hypertension/European Society of Cardiology guidelines [20]. Left ventricular relative wall thickness (LVRWT) was calculated as the ratio of 2×LVPWTd/LVIDd. Cardiac remodeling was defined as LVRWT more than 0.45 without LVH. Concentric LVH was defined as LVMI more than 125 g/m^2^ in men and more than 110 g/m^2^ in women, with LVRWT more than 0.45; eccentric LVH was defined as LVMI more than 125 g/m^2^ in men and more than 110 g/m^2^ in women, with LVRWT less than 0.45. The left atrial volume was measured by the biplane area–length method [21]. Apical 4- and 2-chamber views were obtained to determine the left atrial area and length (from the middle of the plane of the mitral annulus to the posterior wall). The maximal left atrial chamber area and length were measured before mitral valve opening, excluding the left atrial appendage and pulmonary veins. Left atrial volume index (LAVI) was calculated by dividing left atrial volume by body surface area. The raw ultrasonic data were recorded and analyzed offline by a cardiologist, blinded to the other data, using EchoPAC software (GE Medical Systems).

### Assessment of ABI and baPWV

The values of ABI and baPWV were measured by using an ABI-form device (VP1000; Colin Co. Ltd., Komaki, Japan), which automatically and simultaneously measures blood pressures in both arms and ankles using an oscillometric method [22]. ABI was calculated by the ratio of the ankle SBP divided by the arm SBP and the lower value of the ankle SBP was used for the calculation. For measuring baPWV, pulse waves obtained from the brachial and tibial arteries were recorded simultaneously and the transmission time, which was defined as the time interval between the initial increase in brachial and tibial waveforms, was determined. The transmission distance from the arm to each ankle was calculated according to body height. The baPWV value was automatically computed as the transmission distance divided by the transmission time. After obtaining bilateral baPWV values, the higher one was used as representative for each subject. The ABI and baPWV measurements were done once in each patient. The validation of this automatic device and its reproducibility have been previously published [22].

### Assessment of blood pressure

To prevent overestimation and observer bias, interarm difference of blood pressure should be assessed simultaneously at both arms with one or two automated devices [23]. In our study, the bilateral arm blood pressure measurements were done simultaneously and automatically using the ABI-form device. The SBP and diastolic blood pressure (DBP) were measured by an appropriate cuff and the average of SBP and DBP of bilateral arms was used for later analysis.

### Collection of demographic, medical, and laboratory data

Demographic and medical data including age, gender, smoking history, and comorbid conditions were obtained from medical records or interviews with patients. Study subjects were defined as having hypertension if their SBP was ≥140 mmHg or DBP ≥90 mmHg, or antihypertensive medications were prescribed irrespective of blood pressure. The body mass index (BMI) was calculated as the ratio of weight in kilograms divided by square of height in meters. Laboratory data were measured from fasting blood samples using an autoanalyzer (Roche Diagnostics GmbH, D-68298 Mannheim COBAS Integra 400). Serum creatinine was measured by the compensated Jaffé (kinetic alkaline picrate) method in a Roche/Integra 400 Analyzer (Roche Diagnostics, Mannheim, Germany) using a calibrator traceable to isotope-dilution mass spectrometry [24]. The value of estimated glomerular filtration rate was calculated using the 4-variable equation in the Modification of Diet in Renal Disease (MDRD) study [25]. Blood samples were obtained within 1 month of enrollment. In addition, information regarding patient medications including aspirin, angiotensin converting enzyme inhibitors (ACEIs), angiotensin II receptor blockers (ARBs), non ACEI/ARB antihypertensive drugs, and HMG-CoA reductase inhibitors (statins) during the study period was obtained from medical records.

### Statistical analysis

Statistical analysis was performed using SPSS 15.0 for windows (SPSS Inc. Chicago, USA). Data are expressed as percentages, mean ± standard deviation or median (25^th^–75^th^ percentile) for interarm SBP difference and triglyceride. The differences between groups were checked by Chi-square test for categorical variables or by independent t-test for continuous variables. Multiple logistic regression analysis was used to identify the factors associated with an interarm SBP difference ≥10 mmHg. Significant variables in univariate analysis were selected for multivariate analysis. A difference was considered significant if the *P* value was less than 0.05.

## Results

The mean age of the 1120 patients was 60.8±13.7 years. The prevalence of interarm SBP difference ≥10 mmHg was 7.1%. The differences between patients with and without an interarm SBP difference ≥10 mmHg were shown in Table 1. Compared with patients with an interarm SBP difference <10 mmHg, patients with an interarm SBP difference ≥10 mmHg were found to be female, higher prevalence of hypertension, higher mean arterial pressure, higher BMI, higher prevalence of ABI<0.9 (*P* = 0.001), higher baPWV (*P* = 0.044), higher LVMI (*P* = 0.032), lower prevalence of non-LVH (P = 0.012), and higher prevalence of concentric LVH (*P* = 0.018). Table 2 shows the distribution of SBP, DBP, and mean arterial pressure separated by age and gender.

**Table 1:** Comparison of baseline characteristics between patients with and without difference in systolic blood pressure between arms of 10 mmHg or more.

Characteristics	All patients (n = 1120)	Difference <10 (n = 1040)	Difference ≥10 (n = 80)
Difference in systolic blood pressure (mmHg)	3 (2–5)	3 (1–5)	12 (10–14.75)**
Age (year)	60.8±13.7	60.7±13.8	62.1±13.1
Male gender (%)	56.8	58.4	36.3**
Smoking history (%)	14.9	15.1	12.9
Diabetes mellitus (%)	27.5	27.5	27.5
Hypertension (%)	69.0	67.8	85.0*
Coronary artery disease (%)	18.5	18.5	17.9
Cerebrovascular disease (%)	5.5	5.7	3.2
Mean arterial pressure (mmHg)	116.4±17.1	116.0±17.1	120.9±16.8*
Body mass index (kg/m^2^)	26.0±3.9	25.9±3.8	27.9±5.2*
ABI<0.9 (%)	5.4	4.8	13.8*
baPWV (cm/s)	1755.4±448.0	1747.9±452.1	1852.8±379.7*
Laboratory parameters			
Albumin (g/dL)	4.1±0.6	4.1±0.6	4.2±0.4
Fasting glucose (mg/dL)	113.5±40.0	113.1±39.1	119.0±49.3
Triglyceride (mg/dL)	124 (85.75–184.25)	124 (85–185)	125.5 (84–180.75)
Total cholesterol (mg/dL)	192.3±43.8	191.6±44.0	201.1±40.8
Hematocrit (%)	40.4±6.2	40.5±6.3	39.8±5.2
Basline eGFR (mL/min/1.73 m^2^)	58.2±19.6	58.3±19.5	56.3±20.5
Uric acid (mg/dL)	6.8±2.1	6.8±2.1	6.9±2.1
Medications			
Aspirin use (%)	31.7	32.2	25.8
ACEI and/or ARB use (%)	55.2	55.1	57.5
Non-ACEI/ARB antihypertensive drug use (%)	69.2	68.8	73.8
Statin use (%)	19.9	19.9	21.0
Echocardiographic data			
LAVI (ml/m^2^)	35.0±15.2	34.9±15.3	37.1±14.7
LV relative wall thickness	0.39±0.20	0.39±0.20	0.40±0.09
LVMI (g/m^2^)	134.8±45.4	134.0±44.7	145.3±52.9*
LV geometry			
non-LVH	34.1	35.1	21.3*
concentric remodeling	6.3	6.3	7.5
eccentric LVH	45.8	45.6	48.8
concentric LVH	13.8	13.1	22.5*
LVEF (%)	63.1±13.2	63.1±13.2	63.6±13.9
E/A	1.01±0.50	1.01±0.50	0.96±0.50
Ea (cm/s)	8.7±3.4	8.8±3.4	8.0±2.9
E/Ea	9.7±5.2	9.6±5.1	10.5±6.0
E-wave deceleration time (ms)	202.6±63.8	201.9±63.9	212.1±62.4

Abbreviations. ABI, ankle-brachial index; baPWV, brachial-ankle pulse wave velocity; eGFR, estimated glomerular filtration rate; ACEI, angiotensin converting enzyme inhibitor; ARB, angiotensin II receptor blocker; LAVI, left atrial volume index; LVMI, left ventricular mass index; LVH, left ventricular hypertrophy; LVEF, left ventricular ejection fraction; E, transmitral E wave velocity; A, transmitral A wave velocity; Ea, early diastolic mitral velocity.

* *P*<0.05,

** *P*<0.001 compared patients with difference in systolic blood pressure between arms <10 mmHg.

**Table 2:** The distribution of systolic blood pressure (SBP), diastolic blood pressure (DBP), and mean arterial pressure (MAP) separated by age and gender.

Age category	Male (n = 636)			Female (n = 484)		
	SBP (mmHg)	DBP (mmHg)	MAP (mmHg)	SBP (mmHg)	DBP (mmHg)	MAP (mmHg)
<40	133.9±19.9	18.6±13.4	99.0±15.1	130.4±23.2	76.3±14.9	94.3±16.8
40–49	131.3±22.0	81.7±13.3	98.2±15.8	134.9±23.8	79.4±14.5	97.9±16.6
50–59	130.3±19.8	80.0±10.8	96.8±13.8	133.4±17.3	76.4±10.2	95.4±12.1
60–69	136.5±19.7	79.6±11.0	98.6±1.32	139.8±19.5	76.7±10.5	97.7±12.9
70–79	134.8±19.3	74.6±11.5	94.7±12.8	142.6±22.3	74.6±12.0	97.3±14.8
≥80	142.5±22.6	74.7±11.5	97.3±14.5	143.2±24.5	71.0±11.8	95.0±14.9

Compared to men, women were found to have an older age, less smoking history, lower prevalence of coronary artery disease, lower triglyceride, higher total cholesterol, lower hematocrit, lower baseline eGFR and lower uric acid.

Table 3 shows the determinants of an interarm SBP difference ≥10 mmHg in our study patients. In the univariate regression analysis, an interarm SBP difference ≥10 mmHg was found to be significantly associated with female, hypertension, high mean arterial pressure, high BMI, ABI<0.9, high baPWV and high LVMI. We performed two multivariate forward analyses. In the first multivariate analysis (model 1: covariates included gender, hypertension, mean arterial pressure, BMI, ABI<0.9, and baPWV), female, high BMI, ABI<0.9 (odds ratio [OR], 3.670; 95% confidence interval [CI], 1.769 to 7.615; *P*<0.001) and high baPWV (OR, 1.001; 95% CI, 1.000 to 1.001; *P* = 0.002) were independently associated with an interarm SBP difference ≥10 mmHg. In the second multivariate analysis (model 2: covariates included gender, hypertension, mean arterial pressure, BMI, and LVMI), female, hypertension, high BMI, and high LVMI (OR, 1.005; 95% CI, 1.000 to 1.010; *P* = 0.047) were independent factors of interarm SBP difference ≥10 mmHg.

**Table 3:** Determinants of differences in systolic blood pressure between arms ≥10 mmHg in study patients.

Parameter	Multivariate (forward)			
	OR (95% CI)	*P*	OR (95% CI)	*P*
	Model 1		Model 2	
Female *versus* male	2.480 (1.532–4.013)	<0.001	2.773 (1.704–4.514)	<0.001
Hypertension	-	-	1.421 (1.009–2.000)	0.044
Mean arterial pressure (per 1 mmHg)	-	-	-	-
Body mass index (per 1 kg/m^2^)	1.147 (1.086–1.211)	<0.001	1.112 (1.054–1.174)	<0.001
ABI<0.9	3.670 (1.769–7.615)	<0.001		
baPWV (per 1 cm/s)	1.001 (1.000–1.001)	0.022		
LVMI (per 1 g/m^2^)			1.005 (1.000–1.010)	0.047

Values expressed as odds ratio (OR) and 95% confidence interval (CI). Abbreviations are the same as in Table 1.

Covariates in the model 1 included gender, a history of hypertension, mean arterial pressure, body mass index, ABI<0.9 and baPWV.

Covariates in the model 2 included gender, a history of hypertension, mean arterial pressure, body mass index, and LVMI.

## Discussion

In the present study, we evaluated the association of ABI<0.9, baPWV, and LVMI with an interarm SBP difference of 10 mmHg or more. We found that ABI<0.9, high baPWV, and high LVMI were independently associated with an interarm SBP difference of 10 mmHg or more. Other factors, such as female, hypertension, and obesity were also associated with an interarm difference in SBP of 10 mmHg or more.

In our study, the bilateral arm blood pressure measurements were done simultaneously using an automatic device, which might likely reflect the real interarm SBP difference, since previous studies have been based on non-simultaneous blood pressure measurement by a number of different observers using varied sphygmomanometers [8], [26]. Lohmann et al showed simultaneous automatic blood pressure measurement provided smaller and more reproducible interarm SBP difference than conventional non-simultaneous blood pressure measurement and therefore, most likely better estimated a patient's true interarm SBP difference [27]. One meta-analysis showed that the prevalence of a difference in SBP of 10 mmHg or more between arms is roughly doubled when diagnosis is based on one pair of measurements, uses a sequential approach, or uses manual rather than automated measurements [23]. This different measurement method of blood pressure might explain the lower prevalence of an abnormally large interarm SBP difference in our study than in previous studies [8], [26], [28].

A lower ABI is associated with generalized atherosclerosis. There was a significant correlation between ABI and common carotid artery intima-media thickness and the degree of stenosis in the intracranial internal carotid artery and middle cerebral artery [29], [30]. Previous studies had also showed low ABI or ABI<0.9 had correlation with the interarm SBP difference [1], [31], [32]. Our study also revealed that ABI<0.9 and high baPWV were significantly associated with an interarm SBP difference of 10 mmHg or more. Hence, atherosclerosis might represent a causal intermediary between a large interarm SBP difference and poor cardiovascular outcomes.

One important finding of our study was that there was a significant association between high LVMI and the interarm SBP difference of 10 mmHg or more. This finding implies that LVH may be a possible mechanism contributing to the association between an interarm SBP difference of 10 mmHg or more and adverse cardiovascular outcomes. Besides, our study also showed ABI<0.9 and high baPWV were significantly associated with an interarm SBP difference of 10 mmHg or more. These results suggested that an ABI<0.9 and high baPWV might be related to LVH. Previous studies had reported that a low ABI might be related to the interarm SBP difference and LVH [4], [33], [34]. Previous studies demonstrated that the ABI value in the LVH group was significantly lower than that in the non-LVH group and was independently and reversely associated with LVMI [33], [34]. These results suggested that low ABI may be related to LVH. Atherosclerosis directly caused a decrease in blood perfusion in the lower extremities and an increase in arterial wall stiffness, contributing to decreasing ABI and arterial distensibility, and then final progressed to LVH [35]–[37]. Reversely, LVH caused a decrease in cardiac output, which further deteriorated deficiency of blood perfusion of the extremities and promoted the progression of peripheral arterial disease and increased interarm SBP difference. Arterial stiffness is associated with hypertrophy and atherosclerosis within the capacitance arteries that result in an increase in pulse wave velocity (PWV) and consequent alterations in the pressure waveform and increases in systolic and pulse pressure. Alterations in wave reflections combined with increased stiffness may also contribute to LVH [36], [38], [39]. Dellegrottaglie et al. [40] evaluated the association between carotid-femoral PWV and LVMI in 242 patients with chronic kidney disease and found a positive correlation between carotid-femoral PWV and LVMI. In addition, baPWV has been reported to be significantly associated with LVH [41]–[43]. These data may explain the significant association of an interarm SBP difference of 10 mmHg or more with ABI<0.9, high baPWV, and increased LVMI in our patients.

Cardiovascular dysfunction progresses with arterial-cardiac interactions, but the progression of dysfunction differs in speed between the heart and the vessels. In patients with vascular disease, some patients have combined cardiac disease but other patients have only relative pure vascular disease. Hence, patients with vascular disease may not have a higher prevalence of cardiac disease. Left ventricular systolic and diastolic function was mainly determined by cardiac disease such as ischemic heart disease and cardiomyopathy, not mainly by vascular disease. Patients with vascular disease frequently have an increased LVMI [4], [33], [34], [41]–[43]. Our patients with an interarm SBP difference ≥10 mmHg had a more impaired vascular disease indicated by higher prevalence of ABI<0.9 and higher baPWV, which might partially explain the increased LVMI in these patients. In contrast, our patients with an interarm SBP difference ≥10 mmHg might not have a higher prevalence of cardiac disease, which might partially explain the comparable left ventricular systolic and diastolic function such as LVEF, Ea, E/Ea, and LAVI between our patients with an interarm SBP difference ≥10 mmHg and <10 mmHg.

Kimura A et al.'s study had shown that the interarm SBP difference was associated with risk factors for atherosclerosis, such as old age, hypertension, hypercholesterolemia, obesity, and low ABI [32]. The results of our present study were consistent with their findings in that hypertension, high BMI, ABI<0.9, and high baPWV were correlated with an interarm SBP difference of 10 mmHg or more. In Lane D et al.'s and Kimura A et al.'s studies gender was not a significant factor associated with a large interarm SBP difference [26], [32]. However, our study found female gender was an independent risk factor for an interarm SBP difference of 10 mmHg or more. This increased risk might be due to old age and hypercholesterolemia in our female subjects. Further studies are necessary to clarify the association and possible mechanisms between female gender and a large interarm SBP difference.

The majority of our patients were treated chronically with antihypertensive medications. For ethical reasons, we did not withdraw these medications. Hence, we could not exclude the influence of antihypertensive agents on our findings. Moreover, the beat-to-beat variation of blood pressures, ABI, and baPWV during atrial fibrillation is very high. Therefore, we excluded patients with atrial fibrillation. Hence, our results could not be applied in atrial fibrillation. In addition, our patients' blood pressures were not measured using a direct intra-arterial method but through an indirect cuff-oscillometric method, and the readings were made in a supine position rather than in the more standard seated position. Finally, since the subjects of this study are already being evaluated for heart disease, it is susceptible to selection bias and making findings potentially less generalized.

Previous studies reported a relationship between an interarm SBP difference of 10 mmHg or more and cardiovascular events or death [6]–[8]. However, the mechanisms responsible for the association were not fully known. Our results demonstrated that ABI<0.9, high baPWV, and high LVMI were significantly correlated with an interarm SBP difference of 10 mmHg or more. Hence, atherosclerosis and LVH may be possible mechanisms responsible for the association between an interarm SBP difference of 10 mmHg or more and adverse cardiovascular outcomes. Detection of an interarm SBP difference may provide a simple method of detecting patients at increased risk of atherosclerosis and LVH.
